# A Brain Endothelial Cell Caveolin-1/CXCL10 Axis Promotes T Cell Transcellular Migration Across the Blood-Brain Barrier

**DOI:** 10.1080/17590914.2025.2472070

**Published:** 2025-03-10

**Authors:** Troy N. Trevino, Ali A. Almousawi, Remy Martins-Goncalves, Andrea Ochoa-Raya, KaReisha F. Robinson, Genesis L. Abad, Leon M. Tai, Suellen D. Oliveira, Richard D. Minshall, Sarah E. Lutz

**Affiliations:** a Departments of Anatomy and Cell Biology, University of Illinois at Chicago, College of Medicine, Chicago, Illinois, USA; b Anesthesiology, University of Illinois at Chicago, College of Medicine, Chicago, Illinois, USA; c Physiology and Biophysics, University of Illinois at Chicago, College of Medicine, Chicago, Illinois, USA; d Pharmacology and Regenerative Medicine, University of Illinois at Chicago, College of Medicine, Chicago, Illinois, USA

**Keywords:** blood-brain barrier, Caveolin-1, CD4, CXCL10, experimental autoimmune encephalomyelitis, transendothelial migration

## Abstract

The mechanisms that govern whether T cells cross blood-brain barrier (BBB) endothelium by transcellular versus paracellular routes are unclear. Caveolin-1 is a membrane scaffolding and signaling protein associated with transcellular transmigration through the endothelial cytoplasm. Here, we report that the neuroinflammatory chemokine CXCL10 induced transcellular, caveolar transmigration of CXCR3+ CD4+ T cells. Specifically, data revealed that CXCL10-induced transcellular transmigration requires expression of Caveolin-1 and ICAM-1 in brain endothelial cells and of the CXCL10 receptor, CXCR3, and LFA-1 in T cells. Moreover, Caveolin-1 promoted CXCL10 aggregation into brain endothelial cytoplasmic stores, providing a mechanism for activation and recruitment of CXCR3+ T cells to migrate at cytoplasmic locations, distal to cell-cell junctions. Consistent with our *in vitro* data, genetic ablation of Caveolin-1 reduces infiltration of CXCR3+ CD4+ T cells into the CNS in experimental autoimmune encephalomyelitis. Our findings establish a novel mechanism by which brain endothelial cells utilize Caveolin-1 dependent CXCL10 intracellular stores to license T cells for transcellular migration across the blood-brain barrier.

## HIGHLIGHTS

- The neuroinflammatory chemokine CXCL10 specifically promotes transcellular, rather than paracellular, CD4+ T cell transmigration across brain endothelial cells
- Brain endothelial cell Caveolin-1 is required for CXCL10-induced transcellular CD4+ T cell migration
- Brain endothelial cell Caveolin-1 promotes aggregation of CXCL10 into cytoplasmic stores
- CNS infiltration of CD4+ T cells expressing the CXCL10 receptor, CXCR3 is reduced in Caveolin-1 null mice in the EAE model.

## Introduction

The blood-brain barrier (BBB) properties of brain endothelial cells (BECs) regulate leukocyte migration into the brain. Loss of barrier function in BECs can result in an influx of proinflammatory immune cells into the CNS, leading to neuronal damage and adverse outcomes for neurologic function. Identifying mechanisms of leukocyte-endothelial cell crosstalk is important to advance the overall understanding of neuroinflammation.

Leukocytes including CD4+ T cells cross BBB endothelial cells by two routes: paracellular and transcellular. Paracellular migration is defined as diapedesis through the clefts between endothelial cells. BECs restrict paracellular permeability through networks of tight junction (TJs) strands that form a continuous barrier between adjacent cells. In contrast, transcellular migration involves the infiltrating leukocyte diapedesis directly through the endothelial cell cytoplasm (Filippi, 2016; Liebner et al., 2018). Transcellular permeability at the BBB is limited by distinctly low vesicle trafficking in BECs. Paracellular migration is the best studied route of leukocyte-endothelial cell migration in the BBB, whereas transcellular migration is less well mechanistically defined. More thorough mechanistic understanding of how BEC and T cells interact to control transcellular migration is important for a full understanding of BBB permeability.

In transcellular transmigration, Caveolin-1 (Cav-1) forms intracellular vesicles that transfer cargo from the luminal to the abluminal endothelial surface (Jones & Minshall, 2020; Ohi & Kenworthy, 2022; Zimnicka et al., 2016). Suppression of BEC Cav-1 is a key mechanism of BBB maturation, whereas de-repression of Cav-1 induces transcellular BBB permeability (Andreone et al., 2017; Chang et al., 2022; Chow & Gu, 2017; Wang et al., 2020). Cav-1 is upregulated in neuroinflammatory diseases in association with greater T cell infiltration of the CNS (Knowland et al., 2014; Salimi et al., 2020; Trevino et al., 2024; Wu et al., 2016; Zhang et al., 2022), including in experimental autoimmune encephalomyelitis (EAE) (Lutz et al., 2017; Wu et al., 2016). Consistent with a role for Cav-1 in neuroinflammation, Cav-1 deficient mice have less severe demyelination and CNS infiltration of encephalitogenic T cells in EAE (Lutz et al., 2017; Wu et al., 2016). We previously observed Cav-1 dependent transcellular BBB migration is preferentially used by encephalitogenic CD4+ T cells with a Th1 phenotype but not those with a Th17 or a mixed Th1.17 phenotype (Lutz et al., 2017). It is unclear why Th1+ cells preferentially use transcellular routes. However, because Th1 cells have high expression of the interferon-inducible chemokine receptor CXCR3 (Koper et al., 2018; Rahimi & Luster, 2018), it is possible that the CXCR3 receptor is involved in transcellular diapedesis.

CXCL10 is an interferon-inducible chemokine that binds to the CXCR3 receptor. Like Cav-1, CXCL10 is upregulated in regions of BBB permeability (Balashov et al., 1999; Blandford et al., 2023; Heng et al., 2022; Ingelfinger et al., 2024; Koper et al., 2018; Mills Ko et al., 2014; Simpson et al., 2000; Sorensen et al., 2002; Wang et al., 2019; Xu et al., 2020). In fact, the spatial distribution of CXCL10 influences whether infiltrating leukocytes cross the BBB (Muller et al., 2010; Sorensen et al., 2018). The extent to which CXCL10 influences the transcellular/paracellular route of T cell migration, and the underlying mechanism, are not clear.

The goal of this study was to determine the role of CXCL10 in regulating caveolar diapedesis, and whether Cav-1 is important in regulating CXCL10 presentation. Indeed, data indicated CXCL10 stimulation of CXCR3+ T cells enhanced transcellular but not paracellular diapedesis across BECs, dependent on BEC Cav-1. Additionally, Cav-1 expression was required for BEC formation of CXCL10 intracellular stores and was sufficient to induce transcellular diapedesis. Cav-1 also mediated ICAM-1/LFA-1 interactions downstream of CXCR3 to recruit T cells to transcellular migration across BBB endothelia. To validate this mechanistic pathway *in vivo*, we demonstrated that CNS infiltration of CXCR3+ CD4+ T cells was reduced in Cav-1 deficient mice with EAE. Taken together, this work clarifies the mechanism by which brain endothelial CXCL10 and Cav-1 expression facilitate T cell migration across the BBB, bringing unique insights into the understanding of BBB inflammation in autoimmune disease.

## Material & Methods

### Mice

Animal studies were approved by the UIC Animal Care and Use Committee (23-120, 20-160). Wild-type C57Bl/6, Cav-1^-/-^, and CXCR3^-/-^ (Jackson Laboratory 000664, 004585, 005796 respectively) mice were purchased from The Jackson Laboratory. Cav1^-/-^ mice were backcrossed 9 generations by outbreeding to C57Bl/6J. Mice were used between 8-12 weeks of age. Male and female mice were used. Post-hoc tests for effects of biological sex in EAE were conducted (Figure S4).

### Primary Cell Culture

For T cell cultures, splenocytes from C57BL/6 or CXCR3^-/-^ mice were collected 7 days after immunization with MOG_35-55_ in CFA without *Bordetella pertussis* toxin. Splenocytes were cultured in RPMI with 5% fetal bovine serum (FBS), L-glutamine, beta-mercaptoethanol, non-essential amino acids, 20 µg/mL MOG_35-55_, and 1 ng/mL IL-12 (Biolegend 577002) for 3 days to favor Th1 differentiation (Williams et al., 2011). 5 ng/mL IL-2 (Biolegend 575404) was added to media and cells were cultured another 2 days (Lutz et al., 2017).

After 5 days *in vitro,* MojoSort CD4 selection beads (Biolegend 480005) were used to select CD4+ T cells. We used a flow cytometry approach to verify the purification of CD4+ T cells after selection (Fig. S1). For this, we incubated with fixable viability dye (Zombie Green, Biolegend 423111, 1:200) and Fc receptor blockade (anti-mouse CD16/32, Biolegend 101319, 1:200) followed by incubation with antibodies against CD4 (BD Horizon 563151, 1:50) and CXCR3 (Biolegend 126521, 1:100). Cells were analyzed with a Beckman CytoFLEX S flow cytometer. FlowJo software was used for analysis. Sequential gates (Figure S5) were applied to select for leukocyte live populations (based on negativity for the Zombie green viability dye) and singlets (based on forward scatter height:area). Live singlets were gated for CD4. Of CD4+ cells, cells were gated for CXCR3. After CD4 selection, >90% of live cells were CD4+ (Fig. S1A-B). Of the live, CD4+ cell population, ∼50% were CXCR3+ (Fig. S1C).

Primary mouse brain endothelial cells (BECs) were isolated as previously described in detail (Marottoli et al., 2021). Briefly, cerebral cortices were isolated from 3-5 week old mice, minced, mechanically homogenized by passing through a 19 G needle, and digested for 15 minutes at 37 °C with papain (Worthington Biochemical LK003178) and DNase (Worthington Biochemical LK003172). Microvessels were isolated by 25% bovine serum albumin (BSA) gradient centrifugation. Red blood cells were lysed with ammonium-chloride-potassium buffer (150 mM NH_4_Cl, 10 mM KHCO_3_, 0.1 mM Na_2_EDTA). Microvascular endothelial cells were cultured on glass 12-chamber slides (Ibidi 81201) coated with poly-D-lysine (Sigma P7280), fibronectin (Sigma F0895), collagen-I (Sigma C8919), and laminin (L2020) in complete endothelial cell media (Lonza CC-4147, Lonza CC-3156). Inclusion of puromycin (10 μg/mL) (Sigma P8833) during the first 24 hours of culture eliminated non-endothelial cells. Tumor necrosis factor alpha (TNFα) 1 ng/mL (Biolegend 575206) or interferon gamma (IFNγ) 1000 U/mL (Biolegend 575302) was added to BECs 24 hours before T cell migration experiments (Lutz et al., 2017; Martinelli et al., 2014).

Primary CD4+ T cell cultures from MOG_35-55_ immunized C57BL/6 or CXCR3^-/-^ mice were incubated with TNFa-treated primary BECs from C57BL/6 or Cav-1^-/-^ mice in 12-well chamber slides (Ibidi 81201) for transmigration assays. After positive selection for CD4 (MojoSort), T cells were prestimulated with 5 nM CXCL10 for 3 minutes prior to coculturing with mBECs. mBECs were treated with 1 ng/mL TNFα for 24 hours prior to coculture with T cells. In some experiments, mBECs were pretreated with 5 nM methyl-ß-cyclodextrin (Sigma C4555) for 1 hr prior to incubation with CD4+ T cells. In some experiments, CD4+ T cells were pretreated with 40 µg/mL anti-CD18 blocking antibody (Biolegend 101418) for 20 min prior to incubation with mBECs. 50,000 CD4+ T cells were added to each well of 12-well chamber slides containing a confluent monolayer of primary mBECs. CD4+ T cells and mBECs were co-cultured in Hank’s balanced salt solution (HBSS) supplemented with 1% FBS and 5 nM CXCL10 (Biolegend 573604) or vehicle (PBS). CD4+ T cells were allowed to adhere and transmigrate for 60 min at 37 °C in 5 nM CXCL10 or vehicle media under static conditions. After 60 min of coculture, non-adherent CD4+ T cells were washed from mBEC monolayer with HBSS three times and cocultures fixed with cold 4% PFA in PBS in 15 min. Fixed cells were blocked and permeabilized with PBS + 0.01% Tween-20 + 10% BSA for 1 hour before immunostaining for transmigration analysis.

### ICAM-1 Crosslinking

Ten microliters of polystyrene beads (Sigma LB30) were washed 3x with borate buffer (0.1 M boric acid pH 8.5) prior to overnight incubation with 75 ug anti-ICAM1 crosslinking antibody (Thermo 14-0542-85) with rocking at 4° C. Antibody-coated beads were then washed 3x with borate buffer, blocked with 10% BSA in borate buffer, washed 3x with 1% BSA in borate buffer and 2x with 1% BSA in PBS, and stored at 4 C. Prepared anti-ICAM1-coated beads were added to cell culture media at a 1:100 dilution and incubated on mBEC for 5 minutes at 37° C. Bead-containing media was then withdrawn, BEC washed 3x with PBS, fixed, and immunostained.

### Immunofluorescence Imaging and Transmigration Analysis

After positive and negative selection for CD4 and *in vitro* experimentation as indicated, cells were fixed with cold 4% paraformaldehyde in PBS for 15 minutes before staining for immunofluorescence imaging. Fixed cultures were stained with antibodies against CD45 (1:500 Millipore Sigma 05-1416), Zonula occludens-1 (ZO-1, 1:250 Invitrogen 33-9100), and Cav-1 (1:500 Invitrogen PA5-17447). We deployed CD45 (rather than CD4) immunostaining because CD45 yielded the most bright, homogenous labeling of the entire T cell plasma membrane, and allowed the best visualization of invadopodia-like processes. Cross-adsorbed secondary antibodies conjugated to Alexa 488 (Invitrogen A21441), Alexa 594 (Invitrogen A21471), and Alexa 647 (Invitrogen A21463) were used at 1:500. 40X Z-stack images were acquired with Zeiss LSM880 confocal microscope or Leica DMI8 microscope processed with Leica LIGHTNING deconvolution software. To reduce bias, resulting images were coded with randomly generated numbers so the scientist analyzing the data was blind to the conditions. Assessment was fully manual and semi-quantitative, and was conducted by visually assessing each migratory cell one at a time. CD4+ T cells were considered superficially adhered if they were only visible on the apical/luminal side of the mBEC monolayer. T cells were considered migratory if any portion of the cell body or invadapodia-like process protruding on the basolateral/abluminal side of the mBEC monolayer. Migratory cells were categorized as paracellular transmigration if >50% of the cell membrane protrusions colocalized with BEC ZO-1. Migratory cells were categorized as transcellular transmigration if they were embedded in the BEC monolayer with less than 10% of their protrusion surface colocalizing with ZO-1. Ambiguous migrations were excluded from analysis.

### Super Resolution Microscopy

BECs treated for 24h with IFNγ (1000 U/mL) were fixed with 4% paraformaldehyde in PBS for 15 minutes. Fixed cells were blocked and permeabilized with PBS + 0.01% Tween-20 + 10% BSA before immunostaining. Cells were stained with antibodies against CXCL10 (1:500 Abcam ab9938), and against Zona occludens-1 (ZO-1, 1:250 Invitrogen 33-9100) to delineate cell borders. Phalloidin:Alexa594 (1:50 Invitrogen A12381) was used to visualize filamentous actin. Images were acquired with Zeiss LSM880 Airyscan enhanced resolution microscopy at 63x. Z-stacks were collected with axial spacing of 0.5 Nyquist. Images were processed in NIH FIJI (Schindelin et al., 2012). We applied a 3 pixel FFT bandpass filter to reduce digital noise. We used an unbiased strategy for automated quantification of CXCL10 clusters. For this, we used the FIJI Local Maxima plug-in to identify puncta with higher fluorescent intensity than the local background. We used the FIJI “analyze particle” feature to calculate puncta size and fluorescence intensity. Next, we divided cluster number by area to create a density measurement. Area and mean fluorescent intensity of each cluster was averaged for the cells in each field. No regions were omitted from analysis.

### ELISA

Primary Cav-1^+/+^ and Cav-1^-/-^ BECs were isolated and cultured as detailed above in section, *Primary cell culture.* After 5 days in culture, IFNγ (1000 U/mL) (Biolegend 575302) was added to cells in complete endothelial cell media. 24 h later, supernatant was collected. Supernatant was centrifuged for 30 seconds at 2000 x G to pellet any cellular debris before the clarified supernatant was used for ELISA (R&D Systems DY466-05, R&D Systems DY008B). Colorimetric development was detected with a Beckman Coulter DTX880 plate reader. CXCL10 concentration was normalized to total protein concentration in each sample as assessed with Pierce BCA Protein Assay Kit (Thermo 23227).

### EAE

EAE was induced in 8-12 week old mice by subcutaneous injection with 100 µL of inoculant containing 100 µg myelin oligodendrocyte glycoprotein peptide fragment 35-55 (MOG_35-55_ sequence MEVGWYRSPFSRVVHLYRNGK, Thermo Scientific J66557MCR) in PBS with complete Freund’s adjuvant (CFA) containing 100 µg *M. tuberculosis* H37Ra (BD 231141) (Lutz et al., 2012; 2017). Mice received intraperitoneal injections of 100 uL 250 ng/µL toxin from *B. pertussis* (List Biological Laboratories 181) resuspended in PBS at 0- and 2-days post-immunization (DPI). MOG/CFA and pertussis were administered between 3:00 pm and 6:00 pm. Male and female mice were used. Mice were examined for clinical signs of EAE using a 0-5 scale with 0.5 point gradations. 0: no signs, 1: flaccid tail, 2: hind limb paresis, 3: hind limb paralysis, 4: hind and forelimb paralysis, 5: moribund.

### Flow Cytometry *Ex Vivo*

Mice were deeply anesthetized with isoflurane prior to transcardial perfusion with 24 ml ice-cold PBS at a flow rate of 6 ml/minute. Brains and spinal cords were dissected and incubated in ice-cold RPMI until all the dissections were completed. CNS tissues were mechanically homogenized between ground glass slides in cold RPMI. The resulting CNS homogenate was resuspended at a final concentration of 30% Percoll (GE Healthcare P1644) buffered with PBS in a 10 ml volume, and carefully underlaid with 1 ml of 70% Percoll. Homogenate was then centrifuged at 1500 x G for 40 minutes at 4 C without brake. Mononuclear cells were isolated from the interphase of the 30%–70% Percoll gradient. Collected CNS leukocytes were then passed through a 40 micron filter, washed with 5 ml RPMI, centrifuged 1000 x G for 10 minutes at 4° C, and resuspended in PBS. Splenocytes were isolated and homogenized through a 70 µm cell strainer. Single cell suspensions from brain, spinal cord, and spleen were subjected to red blood cell lysis with ammonium-chloride-potassium buffer (150 mM NH_4_Cl, 10 mM KHCO_3_, 0.1 mM Na_2_EDTA) and resuspended in PBS. Fixable viability dye (Zombie Green, Biolegend 423111, 1:200) and Fc receptor blockade (anti-mouse CD16/32, Biolegend 101319, 1:200) were followed by incubation with antibodies against CD45 (Biolegend 103154, 1:100), CD4 (BD Horizon 563151, 1:50), CXCR3 (Biolegend 126521, 1:100), CXCR4 (Biolegend 146505, 1:100), CCR6 (Biolegend 129815, 1:100), CCR2 (Biolegend 150627, 1:100), CD11b (Biolegend 101229, 1:100). Fixation and permeabilization (Biolegend 421403) was followed with an antibody against Cav-1 (Cell Signaling 31411S). Cells were analyzed with a Beckman CytoFLEX S flow cytometer. FlowJo software was used for analysis. Sequential gates (Figure S5) were applied to select for live populations (based on negativity for the Zombie green viability dye), singlets (based on forward scatter height:area), and CD45+ as a pan leukocyte marker. CD45+ cells were gated for CD4. Of CD4+ cells, cells were gated for CXCR3 (Figure 3C). Of the CD45+ cells that were negative for CD4, gates were applied for Ly6G and CD11b (Figure S5).

### Statistical Analysis

Statistical analysis for all experiments was performed in GraphPad Prism v10.2. Results are presented as Mean ± SEM. For pairwise comparisons, F tests were conducted to compare variances; normally distributed data were assessed by unpaired student’s t-test and non-normal or ordinal data were assessed by Mann-Whitney test. For multiple groups, normality was assessed by Brown-Forsythe, followed by one-way ANOVA and Holm-Sidak’s multiple comparisons or non-parametric Kruskall-Wallis and Dunn’s comparisons test. Two variables were assessed by two-way ANOVA with a full effects model and either Fisher’s LSD or Sidak’s multiple comparisons test. Clinical scores of EAE over time were assessed by area under the curve followed by t-test. *p* < 0.05 was considered significant. *p < 0.05 **p < 0.01 ***p < 0.001 ****p < 0.0001.

## Results

### CXCL10 Enhances Adhesion and Transcellular Migration of CD4+ T Cells across BECs

Initially we asked whether CXCL10-CXCR3 influences transcellular permeability. We previously showed that a subset of CD4+ IFNγ+ Th1 cells primarily used the transcellular route to cross the BBB in EAE, whereas IL-17+ subsets used the paracellular route (Lutz et al., 2017). Th1+ cells are characterized by expression of CXCR3, the cognate receptor for CXCL10 (Koper, Kaminska, 2018). This led us to speculate that CXCR3 activation might direct Th1 cells to the transcellular route, especially because the CXCL10-CXCR3 axis correlates with perivascular leukocyte infiltration in MS. Therefore, our first goal was to directly test the idea that CXCR3 activation induces transcellular diapedeses of CD4+ T cell across BECs.

**Figure 1.**
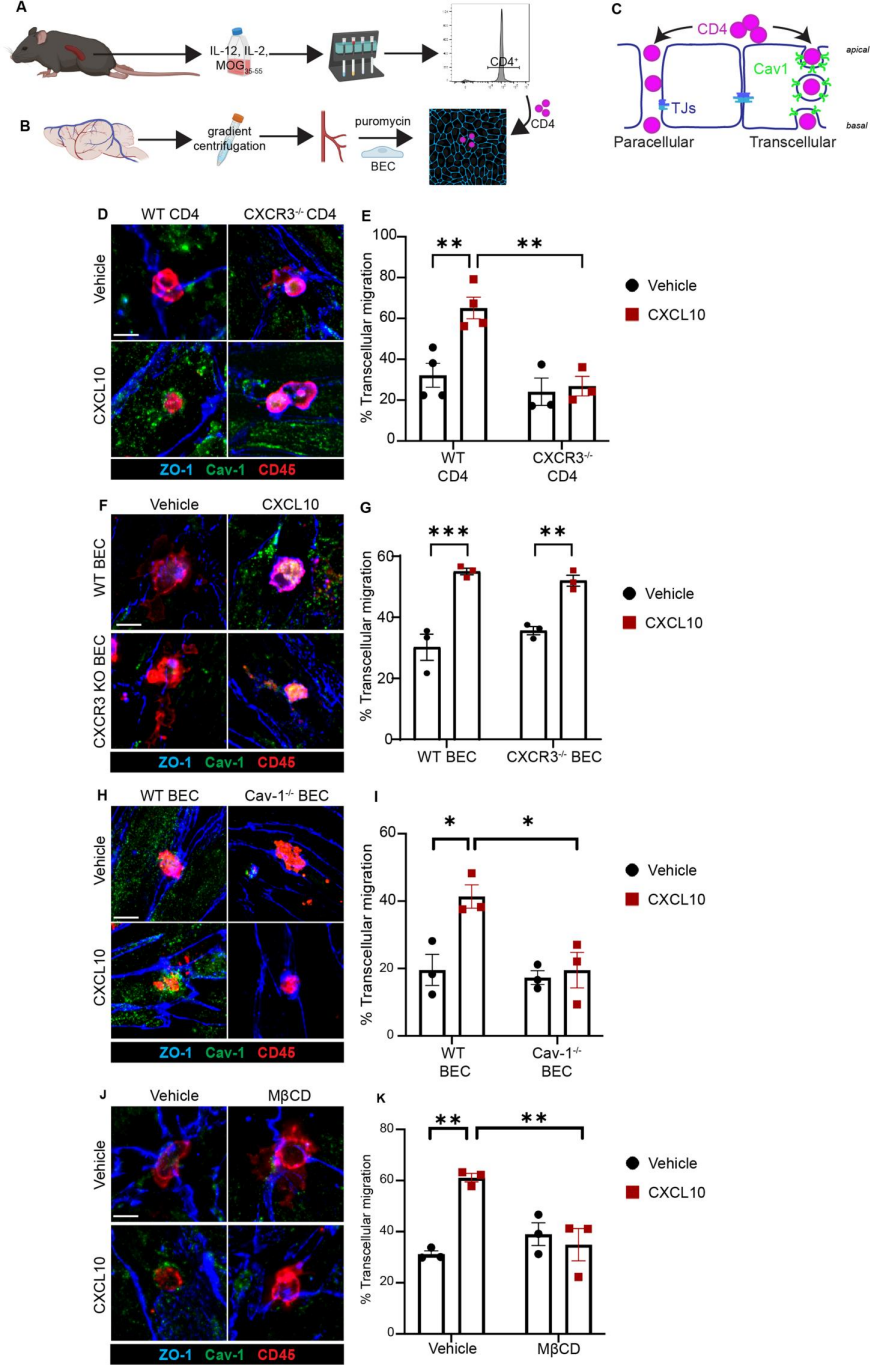
CXCL10-mediated transcellular migration of CD4+ T cells is dependent on endothelial Cav-1 and T cell CXCR3. **A)** Schematic depiction of T cell preparation. Splenocytes from mice with MOG_35-55_ EAE were cultured with MOG_35-55,_ IL-12, and IL-2 cytokines favoring Th1 differentiation. CD4+ T cells were enriched by positive and negative selection on magnetic columns. Histogram of CD4 expression by flow cytometry verified CD4+ T cell enrichment. See also Supplementary Figure 1. **B)** Schematic depiction of mouse brain endothelial cell (BEC) culture preparation. Brains of healthy young adult mice were subject to enzymatic digestion and gradient centrifugation to isolate microvascular cells and cultured with puromycin to eliminate non-endothelial cells. **C)** Schematic depiction of transmigration experiment. CD4+ T cells were applied to cultured BEC and permitted 30 minutes to migrate across the BEC monolayer by paracellular (left) or transcellular (right) diapedesis. Cav-1 indicates Caveolin-1; TJs indicates tight junctions. **D)** Representative immunofluorescent images of WT and CXCR3 KO Th1 CD4+ T cell transmigration across mBECs in the presence of 5 nM CXCL10 or vehicle. Immunostaining was conducted for ZO-1 (blue), Cav-1 (green), and CD45 (red). Examples show T cells engaging with ZO-1+ paracellular junctions in the absence of CXCL10, and T cells engaging with Cav-1+ transcellular sites in the presence of CXCL10. Three to four independent experiments were conducted, with 2-3 technical replicates in each experiment. Scale bar, 10 µm. **E)** Quantification of transcellular migration of WT and CXCR3 KO CD4+ T cells stimulated with 5 nM CXCL10 or vehicle. Two-way ANOVA demonstrated significant effect of CXCL0 [F _(1, 8)_ = 22.41, *p* = 0.0015], T cell CXCR3 genotype [F _(1, 8)_ = 19.3, *p* = 0.0023], and interaction [F _(1, 8)_ = 12.57, *p* = 0.0076]. Fisher’s LSD test revealed that application of CXCL10 increased transcellular transmigration across BEC for WT CD4+ T cells (*p* = 0.0004) but not for CXCR3 KO CD4+ T cells (*p* = 0.4253). Significantly fewer CXCR3 KO T cells than WT CD4+ T cells engaged in transcellular transmigration in response to CXCL10 (*p* = 0.0005). **F)** Representative immunofluorescent images of WT CD4+ T cells transmigrating across WT or CXCR3 KO BEC in the presence of 5 nM CXCL10 or vehicle. Examples show T cells engaging with Cav-1+ transcellular sites in the presence of CXCL10. Three independent experiments were conducted, with 2-3 technical replicates in each experiment. Scale bar, 10 µm. **G)** Quantification of transcellular migration of WT CD4+ T cells across WT or CXCR3 KO BEC stimulated with 5 nM CXCL10 or vehicle. Two-way ANOVA demonstrated significant effect of CXCL0 [F _(1, 8)_ = 68.50, *p* < 0.0001] but not BEC CXCR3 genotype [F _(1, 8)_ = 0.2333,*p* = 0.6420]. Fisher’s LSD test revealed that CXCL10 increased transcellular transmigration across WT BEC (*p <* 0.0001) and across CXCR3 KO BEC (*p* < 0.0016). No difference was noted between WT BEC and CXCR3 KO BEC upon stimulation with CXCL10 (*p* = 0.4151). Three independent experiments were conducted, with 2-3 technical replicates in each experiment. **H)** Representative immunofluorescent images of WT CD4+ T cells transmigrating across WT or Cav-1 KO BEC in the presence of 5 nM CXCL10 or vehicle. Examples show T cells at transcellular sites of diapedesis across WT BEC and at paracellular sites on Cav-1 KO BEC. Three independent experiments were conducted, with 2-3 technical replicates in each experiment. Scale bar, 10 µm. **I)** Quantification of transcellular migration of WT CD4+ T cells transmigrating across WT or Cav-1 KO BEC in the presence of 5 nM CXCL10 or vehicle. Two-way ANOVA demonstrated significant effect of CXCL10 [F _(1, 8)_ = 8.795, *p* = 0.0180], BEC Cav-1 genotype [F _(1, 8)_ = 8.929,*p* = 0.0174], and interaction [F _(1, 8)_ = 5.873, *p* = 0.0416]. Fisher’s LSD test revealed that CXCL10 increased transcellular transmigration across WT BEC (*p* < 0.0052) but not across Cav-1 KO BEC (*p* = 0.7114). Significantly less CD4+ T cell transcellular transmigration was observed across Cav-1 KO BEC than across WT BEC in response to CXCL10 (*p* < 0.005). **J)** Representative immunofluorescent images of WT CD4+ T cells transmigrating across WT BEC treated with vehicle or methyl-beta cyclodextrin (MβCD) to disrupt caveolae, in the presence of 5 nM CXCL10 or vehicle. Examples show T cells engaged in paracellular transmigration with MβCD. Three independent experiments were conducted, with 2-3 technical replicates in each experiment. Scale bar, 10 µm. **K)** Quantification of transcellular migration of WT CD4+ T cells transmigrating across WT BEC with or without MβCD, in the presence of 5 nM CXCL10 or vehicle. Two-way ANOVA demonstrated significant effect of CXCL10 [F _(1, 8)_ = 10.39, *p* = 0.0122], approached significance for effect of MβCD [F _(1, 8)_ = 5.303, *p* = 0.0503], and significant effect for interaction [F _(1, 8)_ = 18.09, *p* = 0.0028]. Fisher’s LSD test revealed that CXCL10 increased transcellular transmigration across BEC in the absence of MβCD (*p* = 0.007) but not in the presence of MβCD (*p* = 0.4875). Significantly less CD4+ transcellular transmigration occurred in response to CXCL10 after application of MβCD (*p* = 0.0017).

Primary MOG_35-55_-specific CD4+ T cells were isolated from WT or CXCR3 deficient (CXCR3^-/-^) mice, differentiated into Th1-like cells by culturing with IL-12, IL-2, and MOG_35-55_ for 5 days, and sorted by positive selection to CD4+ (Figure 1A-B). CXCR3^+/+^ and CXCR3^-/-^ CD4+ T cells were applied to a monolayer of primary BECs in the presence of 5 nM CXCL10. We identified 5 nM CXCL10 as the optimum concentration for eliciting transcellular migration (Fig. S2A). After 1-hour, non-adherent T cells were washed away, cultures were fixed, and T cells adherent to BEC monolayers were visualized by immunofluorescence. In initial studies we observed that CD4 immunostaining did not fully and uniformly label leading edge processes. We therefore deployed CD45 immunostaining because it enabled more sensitive visualization of sites of T cell:BEC interaction. Importantly, flow cytometry experiments confirmed that cultures used in these studies were >90% CD4+ after positive selection (Fig. S1). Adherent CD4+ T cells were therefore quantified by immunostaining for the pan-leukocyte marker CD45 (Figure 1D, F, H, J). As expected, adhesion of CXCR3^+/+^ but not CXCR3^-/-^ CD4+ T cells to BECs was enhanced with CXCL10 (Fig. S2B). Z-stacks were acquired to analyze the diapedesis route (Lutz et al., 2017). Transcellularly migrating cells were frequently flanked by endothelial Cav-1 (Figure 1D). We found that CXCL10 stimulation significantly increased transcellular CD4+ T cell interactions with BECs and that this increase was abolished in CXCR3^-/-^ CD4+ T cells (Figure 1D-E). BEC can also express CXCR3, so we asked if CXCL10 acting on BEC CXCR3 influenced transcellular transmigration. Genetic deficiency of BEC in CXCR3 did not affect CXCL10-stimulated transcellular migration of CD4+ T cells (Figure 1F-G), suggesting that CXCL10 increases transmigration by activating CXCR3 on T cells rather than on BEC. These data suggest that CXCL10 primes the CD4+ T cells for transcellular migration across the BBB by activation of T cell CXCR3.

### CXCL10-Mediated, Cav-1 Dependent Transcellular Migration of CD4+ T Cells

Next, we asked if CXCL10-induced transcellular diapedesis requires BEC Caveolin-1. To interrogate the role of Cav-1 in transcellular migration of CXCR3+ CD4+ T cells across BECs, we applied MOG_35-55_-specific CD4+ T cells to Cav-1^+/+^ or Cav-1^-/-^ BECs in the presence of 5 nM CXCL10. We found that transcellular migration of CXCL10-stimulated CD4+ T cells was absent in Cav-1^-/-^ BECs (Figure 1H-1). Endothelial Cav-1 deficiency did not prevent CXCL10-mediated CD4+ T cell adhesion to BECs (Fig. S2C). This indicates that CXCL10 stimulation of transcellular migration of CD4+ T cells across the BBB involves endothelial cell Cav-1 expression.

Cav-1 is a membrane anchored protein that forms complexes which regulate signal transduction and endocytic vesicle trafficking (Ohi & Kenworthy, 2022). These complexes, called caveolae, form within cholesterol-enriched membrane microdomains in cells expressing Cav-1. Cyclodextrins are cyclic oligosaccharides commonly used as chelating agents. Methyl-β-cyclodextrin (MβCD) binds to cholesterol and free fatty acids in plasma membranes and has been widely used as a pharmacologic approach to disrupt caveolae (Castagne et al., 2010). Here, we utilized MβCD to further investigate the contribution of BEC caveolae in transcellular migration of CD4+ T cells. BECs were treated with 10 mM MβCD for 1 hour prior to coincubation with MOG_35-55_-specific CD4+ T cells and 5 nM CXCL10. In agreement with data obtained in our targeted genetic approach (Figure 1H-1), MβCD disruption of caveolae in BECs inhibited transcellular diapedesis of CD4+ T cells in the presence of CXCL10 (Figure 1J-K). MβCD also did not inhibit CXCL10-stimulated adhesion (Fig. S2D). Together, data generated using genetic and pharmacologic approaches to disrupt endothelial Cav-1 expression or functional caveolae suggests that application of CXCL10 to the coculture medium induced transcellular diapedesis of CD4+ T cells that is dependent on endothelial Cav-1 expression.

### BEC Cav-1 Organizes CXCL10 into Intracellular Stores

Next, we focused on whether Cav-1 could also influence upstream steps in transcellular migration such as bioavailability of endogenously produced chemokines. Endothelial CXCL10 is specifically linked to T cell transmigration across blood vessels outside of the nervous system (Schoppmeyer et al., 2022; Shulman et al., 2011).; Furthermore, endocytic activity of Cav-1 disrupts the apicobasal distribution of CXCL12 in BEC (Cruz-Orengo et al., 2014). Therefore, we asked whether Cav-1 could regulate BEC production and presentation of CXCL10.

**Figure 2.**
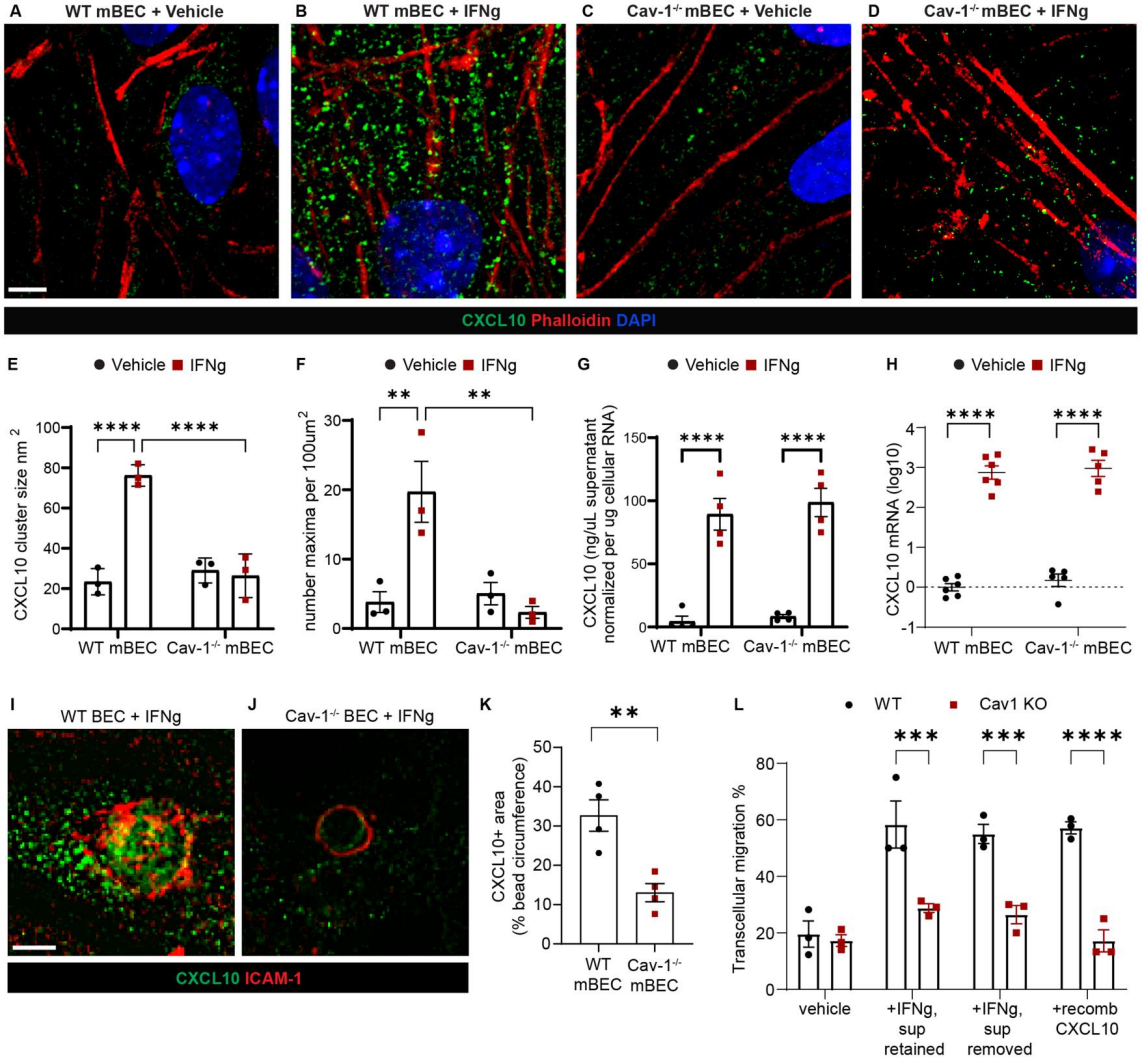
Endothelial Cav-1 dependent intracellular stores of CXCL10 induce transcellular transmigration. **A-D)** Representative super-resolution immunofluorescence images of intracellular CXCL10 (green) in BEC stimulated with vehicle or with IFNγ for 16 hours. Phalloidin (red) marks filamentous actin. DAPI (blue) indicates nucleic acid. Scale bar 2.5 µm. Images show pronounced clusters of cytoplasmic CXCL10 in IFNγ treated WT BEC. **E)** Quantification of mean CXCL10 cluster size. Two-way ANOVA demonstrated significant effect of IFNγ [F _(1, 8)_ = 33.29, *p* = 0.0004], BEC genotype for Cav-1 [F _(1, 8)_ = 25.91, *p* = 0.0009], and interaction [F _(1, 8)_ = 40.72, *p* = 0.0002]. Fisher’s LSD test revealed that IFNγ induced CXCL10 cluster expansion in WT BEC (*p* < 0.0001) but not in Cav-1 KO BEC (*p* = 0.677). CXCL10 cluster size was significantly smaller in IFNγ-stimulated Cav-1 KO BEC than in WT BEC (*p* < 0.0001). Three independent experiments were conducted, with 3 technical replicates in each experiment. **F)** Quantification of number of CXCL10 clusters detected per 100 square microns. Two-way ANOVA demonstrated significant effect of IFNγ [F _(1, 8)_ = 6.983,*p* = 0.0296], BEC genotype for Cav-1 [F _(1, 8)_ = 10.48,*p* = 0.0119], and interaction [F _(1, 8)_ = 13.88, *p* = 0.0058]. Fisher’s LSD test revealed that IFNγ increased CXCL10 cluster density in WT BEC (*p* = 0.002) but not in Cav-1 KO BEC (*p* = 0.4658). CXCL10 density was significantly lower in IFNγ stimulated Cav-1 KO BEC than in WT BEC (*p* = 0.0012). Three independent experiments were conducted, with 3 technical replicates in each experiment. **G)** Quantification of CXCL10 in supernatant of BEC stimulated with IFNγ, as assessed by ELISA. To control for any variation in BEC density, CXCL10 concentration in the supernatant is normalized according to BEC total mRNA. Two-way ANOVA demonstrated significant effect of IFNγ on CXCL10 secretion [F _(1, 12)_ = 102.1, *p* < 0.0001]. BEC genotype for Cav-1 did not effect CXCL10 secretion [F _(1, 12)_ = 0.5916, *p* = 0.4567]. Two independent experiments were conducted, comprising a total of four biological replicates. **H)** Quantification of CXCL10 mRNA in lysate of BEC stimulated with IFNγ, as assessed by qRT-PCR. Two-way ANOVA demonstrated significant effect of IFNγ stimulation on CXCL10 mRNA [F _(1, 18)_ = 330.1, *p* < 0.0001]. BEC genotype did not effect CXCL10 mRNA [F (1, 18) = 0.7960, *p* = 0.3841]. Two independent experiments were conducted, comprising a total of 5-6 biological replicates. **I-J)** Representative immunofluorescence images of CXCL10 clusters (green) in BEC stimulated with IFNγ and incubated with polystyrene beads coated with ICAM-1-cross-linking antibody. The ring-shaped pattern of immunofluorescence for ICAM-1 (red) reveals the recruitment of ICAM-1 around the beads coated with ICAM-1-binding antibody. The representative image demonstrates enrichment of CXCL10 in the area of high ICAM-1 crosslinking in WT BEC. **K)** Quantification of CXCL10 density at sites of ICAM-1 enrichment in BEC incubated with polystyrene beads coated with ICAM-1-cross-linking antibody. Unpaired t-test revealed that as compared with WT BEC, Cav-1^-/-^ BEC have reduced area fraction of CXCL10 immunopositive pixels in ICAM-1+ area (*p* = 0.0053). **L)** Transcellular transmigration of CD4+ T cells across BEC with or without supernatant removal. Two-way ANOVA demonstrated significant effect for BEC Cav-1 genotype [F _(1, 16)_ = 72.39, *p* < 0.0001], treatment [F _(3, 16)_ = 14.76, *p* < 0.0001], and interaction [F _(3, 16)_ = 7.402, *p* = 0.0025]. Sidak’s multiple comparisons test revealed that WT BEC stimulated with IFN**γ** supported significantly more transcellular transmigration that did WT BEC without stimulation (*p* < 0.0001). In fact, the extent of transcellular transmigration was not different between IFN**γ** stimulated WT BEC and unstimulated WT BEC treated with CXCL10 (*p* > 0.9999), suggesting that IFN**γ**-stimulated BEC produce sufficient quantities of chemokine to induce transcellular transmigration. Importantly, removal of the supernatant from IFN**γ** stimulated WT BEC did not hamper the transcellular transmigration of T cells (*p* = 0.9948), consistent with a contribution of non-secreted, cytoplasmic CXCL10 to promote transcellular transmigration across WT BEC. Furthermore, no significant transcellular transmigration was induced in Cav-1 KO BEC stimulated with IFN**γ** (*p* = 0.3486), stimulated with IFN**γ** and supernatant removed (*p* = 0.5946), or upon addition of recombinant CXCL10 (*p* = >0.9999).

CXCL10 was analyzed in primary Cav-1^+/+^ and Cav-1^-/-^ BECs treated overnight with interferon gamma (IFNγ), the canonical activator for CXCL10 production (Koper, Kaminska, 2018). Abundance and size of cytoplasmic CXCL10 intracellular stores (Schoppmeyer et al., 2022; Shulman et al., 2011) were interrogated in super resolution confocal Z-stacks (Figure 2A-F). In Cav-1^+/+^ BECs, IFNγ increased the size (Figure 2E) and number (Figure 2F) of CXCL10 stores. In contrast, there was no such expansion of CXCL10+ size or frequency in IFNγ-treated Cav-1^-/-^ BECs (Figure 2C-F). These data indicate that Cav-1 promotes the organization of CXCL10 into BEC cytoplasmic stores.

One explanation for sparse induction of CXCL10 intracellular stores by exposure to IFNγ, could be a requirement for Cav-1 in secretion or synthesis of CXCL10. To test this, we first asked if less cytoplasmic CXCL10 in the Cav-1^-/-^ BEC could be due to greater secretion into the supernatant. We collected supernatant from Cav-1^+/+^ and Cav-1^-/-^ BECs for CXCL10 measurement by ELISA. IFNγ induced CXCL10 secretion from Cav-1^+/+^ and from Cav-1^-/-^ BEC (Figure 2G). Next, we investigated synthesis. IFNγ-induced CXCL10 mRNA levels were similar in Cav-1^+/+^ and Cav-1^-/-^ BEC, as revealed by QPCR (Figure 2H). These data indicated that Cav-1 is not required for CXCL10 production.

Transcellular T cell migration is promoted when T cells push into endothelial cell transmigratory “cups” enriched in Cav-1 and leukocyte adhesion molecules including ICAM-1 (Carman et al., 2007; Carman & Springer, 2004; Millan et al., 2006; Schoppmeyer et al., 2022). Therefore, we asked if Cav-1 contributes to the local concentration of CXCL10 at sites of ICAM-1 clustering. We applied polystyrene beads coated with ICAM-1 crosslinking antibody to Cav-1^+/+^ and Cav-1^-/-^ BEC and conducted immunostaining and microscopy for CXCL10. Indeed, less CXCL10 was recruited to the ICAM-1 crosslinking beads in Cav-1^-/-^ BEC than in Cav-1^+/+^ BEC (Figure 2I-K). These data indicate that Cav-1 contributes to the aggregation and delivery of CXCL10 to sites of ICAM-1 clustering.

Given these observations, we next asked whether Cav-1 dependent stores of endothelial CXCL10 were sufficient to induce CD4+ T cell transcellular migration. As before, CXCL10 production was induced with IFNγ in Cav-1^+/+^ and Cav-1^-/-^ BEC. The next day, supernatant (containing secreted CXCL10) was withdrawn and replaced with fresh media immediately prior to co-incubation with CD4+ T cells. Strikingly, transcellular transmigration occurred at similar rates when supernatant was removed from Cav-1^+/+^ but not Cav-1^-/-^ BEC, suggesting that CXCL10 cytoplasmic stores are sufficient to induce transcellular transcytosis (Figure 2L). In fact, the extent of transcellular transmigration induced in cytokine-stimulated Cav-1^+/+^ BEC was similar to the cultures in which exogenous CXCL10 was added as a positive control (Figure 2L). These data indicate that Cav-1 dependent CXCL10 intracellular stores are sufficient to induce transcellular transmigration of CD4+ T cells.

### CD4+ T Cell Transcellular Migration across BECs is Dependent on LFA-1/ICAM-1 Signaling

Some chemokine receptor signaling leads to inside-out activation of integrins. For CXCR3, stimulation with CXCL10 promotes activation of LFA-1, an important integrin in T cell migration (Ngwenyama et al., 2019; Prizant et al., 2021; Wen et al., 2022). We investigated if Cav-1 influences CXCL10-mediated transcellular BEC migration by effects on CD4+ T cell LFA-1 interaction with endothelial cell ICAM-1. MOG_35-55_-specific CD4+ T cells were treated with 40 ng/mL integrin β_2_ blocking antibody (anti-CD18) to inhibit LFA-1 prior to addition of T cells to BECs, in the presence of 5 nM CXCL10. We found that blocking LFA-1 with anti-CD18 antibody, but not an isotype control antibody, impeded adhesion (Fig. S2E) and transcellular migration (Fig. S3A-B) of CXCL10-activated MOG_35-55_-specific CD4+ T cells across BECs. Together with previous reports, this data suggests CXCL10/CXCR3 T cell activation promotes transcellular, Cav-1 dependent diapedesis through BEC by a mechanism involving LFA-1/ICAM-mediated signaling.

### Cav-1 Deficient Mice Have Reduced CXCR3+ T Cell Infiltration in the CNS during EAE

**Figure 3.**
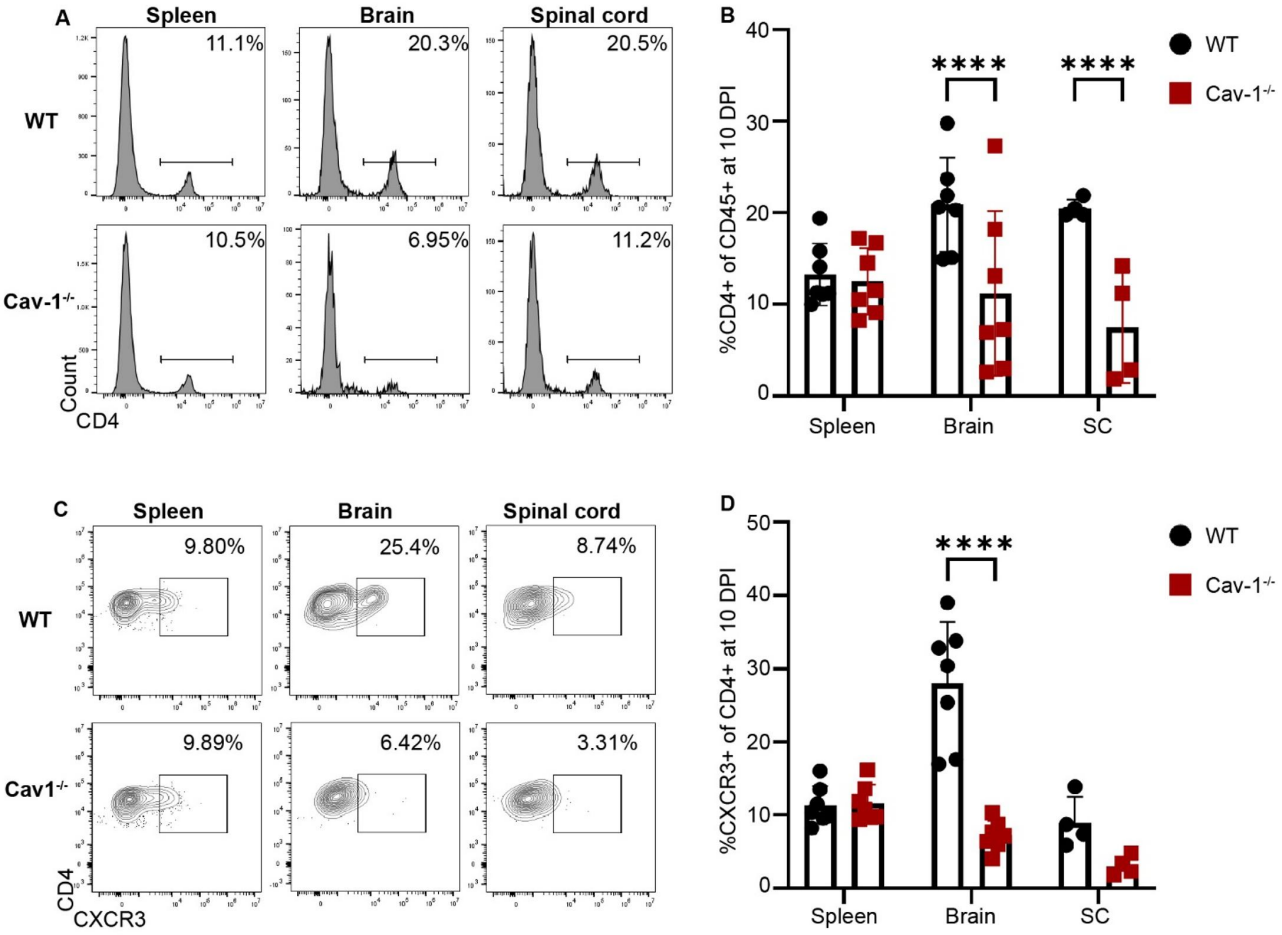
Cav-1 facilitates CXCR3+ CD4+ T cell entrance to the CNS in EAE. **A)** Flow cytometric histograms representing CD4+ T cell frequency in spleen, brain, and spinal cord (SC) of WT and Cav-1 KO mice at 10 days post immunization with MOG_35-55_ EAE. Cells were gated on viable dye exclusion and CD45^hi^. **B)** Bar graph representing CD4+ T cell frequency in the spleen and CNS of mice with EAE. Two-way ANOVA demonstrated significant effect of genotype [F _(1, 18)_ = 43.76, *p* < 0.0001] and genotype*organ interaction [F _(2, 18)_ = 9.682, *p* = 0.0014]. Sidak’s multiple comparisons test revealed that Cav-1 KO mice with EAE had significantly less CD4+ T cells than did WT mice with EAE in the brain (*p* < 0.0001) and in the spinal cord (*p* < 0.0001). **C)** Flow cytometric histograms representing CXCR3+ CD4+ T cell frequency in spleen, brain, and spinal cord of WT and Cav-1 KO mice at 10 days post immunization with MOG_35-55_ EAE. Cells were gated on viable dye exclusion, CD45^hi^, and CD4^+^. **D)** Bar graph representing CXCR3+ CD4+ T cell frequency in the spleen and CNS of mice with EAE. Two-way ANOVA demonstrated significant effect of genotype [F _(1, 18)_ = 23.85, *p* = 0.0001], organ [F _(2, 18)_ = 11.06, *p* = 0.0007], and genotype*organ interaction [F _(2, 18)_ = 9.256, *p* = 0.0017]. Sidak’s multiple comparisons test revealed that Cav-1 KO mice with EAE had significantly less CXCR3+ CD4+ T cells in the brain than did WT mice with EAE (*p* < 0.0001).

We next asked if Cav-1 regulates CNS infiltration of CXCR3+ T cells *in vivo*. To do this, we induced MOG_35-55_ EAE in Cav-1^+/+^ and Cav-1^-/-^ mice. We previously reported that female Cav-1^-/-^ mice have less severe clinical signs of EAE concordant with less extensive demyelination and selective deficiency in IFNγ+ CD4+ T cell infiltration of the CNS (Lutz et al., 2017). First, we replicated previous reports (Lutz et al., 2017; Wu et al., 2016) that Cav-1^-/-^ mice had less severe clinical signs of EAE (Fig. S4A-C). We then assessed male and female mice and determined that biologic sex did not influence neurological expression of disease (Fig. S4D-I). Next, we assessed leukocyte infiltration of the CNS by flow cytometry (gating strategy depicted in Fig S5). CD4+ T cells were reduced in the CNS of Cav-1^-/-^ mice as compared to Cav-1^+/+^ mice during the onset of signs of EAE, at 10 days post immunization (DPI) (Figure 3A-B). Further characterization revealed that CXCR3+ CD4+ T cells were specifically reduced in the brains of Cav1^-/-^ mice at the onset of disease (Figure 3C-D). Similar reduction of CXCR3+ CD4+ T cells in the Cav-1^-/-^ brain was also observed at the peak of clinical signs of EAE, at 17DPI (Fig. S6). These data are consistent our previous report of fewer IFNγ+ CD4+ T cells, and less demyelination, in the spinal cords of Cav-1^-/-^ mice with EAE. CXCR3+ expression was unchanged in splenic CD4+ T cells in Cav1^-/-^ indicating that Cav-1 is not required for the stability of CXCR3+ CD4+ T cells in the periphery (Figure 3C-D and Fig. S5C-D). Neutrophil infiltration was similar in Cav-1^+/+^ and Cav-1^-/-^ CNS (Fig. S7A). Monocyte infiltration into the spinal cord but not the brain was reduced in Cav-1^-/-^ compared to Cav-1^+/+^ mice at EAE onset (Fig. S7B). Cav-1 deficiency did not significantly alter CXCR3+ neutrophil or monocyte CNS infiltration (Fig. S7C-D).

We also asked if Cav-1 regulates BBB migration of other subsets of CD4+ T cells by probing CNS CD4+ T cells for expression of a subset of chemokine receptors associated with Th17 and regulatory T cell phenotype (Heng et al., 2022). We found no difference in CCR6, CXCR4, and CCR2 expressing CD4+ T cells in the CNS or periphery associated with Cav-1 deficiency during EAE (Fig. S7E-F). Together, these data demonstrate that Cav-1 is selectively important for the CNS infiltration of CXCR3+ CD4+ T cells across the BBB.

## Discussion

Outside of the nervous system, endothelial cells regulate the infiltration of leukocytes into tissue beds by transcellular and paracellular routes; this is enhanced by chemokines. Both of these routes of leukocyte extravasation are suppressed at the BBB by the actions of BEC. This is important because excessive chemokine-induced entry of leukocytes into the brain contributes to neuroinflammation and neurological deficits in diseases including multiple sclerosis. Therefore, understanding the mechanisms by which BEC regulate chemokine-induced leukocyte extravasation is important. However, transcellular transmigration has been relatively less well characterized than paracellular transmigration. It is established that transcellular permeability increases in inflammation and injury in correlation with impaired neurologic performance, and that Cav-1 is a central component of the transcellular permeability pathway. Indeed, we and others have previously reported that Cav-1^-/-^ mice are partially protected from EAE in association with less T cell infiltration, neuroinflammation, and demyelination (Lutz et al., 2017; Wu et al., 2016). However, the mechanisms by which endothelial Cav-1 contributes to transcellular transmigration at the BBB, and the contribution of BEC-produced chemokines to this process, are unclear. Our study provides evidence that CXCL10 enhances transcellular BBB transmigration of CXCR3+ T cells in a Cav-1 dependent manner. Moreover, we show that IFNγ-stimulated BEC produce sufficient quantities of CXCL10 to induce transcellular migration, and that the bioavailability of the resulting BEC CXCL10 pool is regulated by Cav-1. These data were supported by *in vivo* studies showing reduced CNS infiltration of CXCR3+ CD4+ T cells in Cav-1^-/-^ mice with EAE, which provides mechanistic insight into how CXCL10 can impact T cell transcellular transmigration at the BBB.

Previous studies have also found that CXCL10/CXCR3 ligation promotes LFA-1/ICAM-1 activation and adhesion (Ngwenyama et al., 2019; Prizant et al., 2021), and that transmigratory cups enriched in Cav-1, chemokines, and leukocyte adhesion molecules induce transcellular transmigration by close interactions with leukocyte invadopodia-like processes (Barreiro et al., 2002; Carman & Springer, 2004, 2008, Heemskerk et al., 2016; Martinelli et al., 2014; Shulman et al., 2011; van Steen et al., 2023). Furthermore, BEC chemokine localization is at least indirectly influenced by Cav-1 (Cruz-Orengo et al., 2014), and chemokine presentation by activated endothelial cells may promote transcellular (rather than paracellular) diapedesis (Abadier et al., 2015; Marchetti et al., 2022). Brain endothelial cell production of CXCL10 has been described (Chen et al., 2023; Sorensen et al., 2018). Our study expands on these findings by showing that Cav-1 regulates CXCL10 bioavailability, and that CXCL10 enhances transcellular migration in a mechanism involving Cav-1- and ICAM-1-dependent internalization. Thus, the findings of this study provides mechanistic details that have hitherto been lacking.

Our studies address the important topic of how endothelial cell intrinsic signals, leukocyte signals, and the interaction between them influences leukocyte transendothelial migration. According to the tenertaxis model, crawling leukocytes probe the endothelium for paracellular-versus-transcellular “sites of least resistance” where they will diapedese (Martinelli et al., 2014). This is supported by data showing that BEC, with their extensive network of stable tight junction strands, are more resistant to paracellular leukocyte diapedesis than are peripheral EC (Martinelli et al., 2014). Recent work has further established that endothelial junctions with rapid actin dynamics become diapedesis hotspots (Arts et al., 2021). Thus, endothelial-intrinsic features regulate the method and extent of diapedesis. Relatively less attention has been devoted to leukocyte-intrinsic signals (such as CXCR3 levels) that contribute to this process. Our work refines the tenertaxis model by establishing that the Cav-1/CXCL10 axis promotes transcellular diapedesis of CXCR3+ Th1+ T cells. A similar mechanism might exist for other chemokine/leukocyte combinations. This provides the foundation for future studies to determine which chemokine/integrin combinations control transcellular migration patterns of other leukocytes.

An important future direction is to identify the downstream mechanism by which endothelial CXCL10 is released to the leukocytes. Endothelial cells outside of the CNS promote T cell migration by the regulated release of intracellular chemokine stores at locations of intimate leukocyte-endothelial interaction, creating a microenvironment favorable for transmigration (Alon & Shulman, 2011; Marchetti et al., 2022; Schoppmeyer et al., 2022; Shulman et al., 2009; 2011). In peripheral vascular endothelial cells, ICAM-1 clustering induced synapse-like regulated release of vesicular stores of CXCL10, CXCL8, and CXCL1 and promoted transcellular T cell diapedesis (Schoppmeyer et al., 2022). Moreover, CXCL10 clusters were more effective than monomers at inducing transendothelial T cell migration (Campanella et al., 2006). In our study, upon depletion of CXCL10-containing supernatant, the residual intra-endothelial CXCL10 clusters were sufficient to initiate transcellular transmigration. Further experimentation is required to define the mechanistic role of Cav-1 in stimulated release of BEC CXCL10 intracellular stores, and to determine whether caveolar-dependent signals including heterogenous chemokine stores encountered by extravasating T cells can have lasting effects on pro- or anti-inflammatory behavior within the target tissue (Karin, 2020).

These findings should be considered in the context of several limitations. We are limited in the extent that we can draw conclusions on the contribution of endothelial cell Cav-1 to T cell migration *in vivo*; the relative contribution of endothelial cell Cav-1 as opposed to expression in other cell type is not resolved. Indeed, Cav-1 is widely expressed including in T cells and may contribute to vascular dysfunction and neuroinflammation by promoting T cell receptor signaling (Borger et al., 2017; Schönle et al., 2016; Tomassian et al., 2011) and chemotaxis (Oyarce et al., 2017; Reese et al., 2022). Future studies using inducible brain-endothelial cell-targeted deletion and/or reconstitution of Cav-1 would better define the specific role of endothelial Cav-1. Moreover, the Cav-1^-/-^ mouse has phenotypic complications including vascular hyperpermeability (Miyawaki-Shimizu et al., 2006; Razani et al., 2001; Schubert et al., 2002; Sun et al., 2009), vasodilation (Chen et al., 2018), and decreased cerebrovascular density (Head et al., 2010), whereas we and others have reported vascular hypopermeability in the absence of Cav-1 due to decreased junction turnover (Marchiando et al., 2010; Song et al., 2007; Stamatovic et al., 2009; Trevino et al., 2024). Thus, the attenuation of EAE in the Cav-1^-/-^ mouse may also be influenced by structural and functional changes in the vasculature prior to the induction of disease.

We propose that for CD4+ Th1 cells expressing CXCR3, CXCL10 ligation promotes LFA1-dependent interaction with endothelial cells at sites of Cav-1, thereby facilitating Cav-1 dependent transcellular transmigration. Our *in vitro* and *in vivo* data is consistent with this possibility. This is also consistent with known roles for CXCR3 in promoting Th1 cell polarization, integrin activation and cytoskeletal remodeling (Kendirli et al., 2023; Ngwenyama et al., 2019). We also considered an autocrine pathway, where brain endothelial cell secretion of CXCL10 acts on brain endothelial cell CXCR3 to increase permeability. This appears not to be a major mechanism, because there was no statistical difference in the extent of CXCL10-induced transcellular migration across brain endothelial cells cultured from CXCR3^-/-^ or CXCR3^+/+^ mice (Figure 2G). Importantly, brain endothelial cells are only one of several sources of CXCL10. CXCL10 is also robustly secreted by non-endothelial cells of the neurovascular unit (Fife et al., 2001; Koper, Kaminska, 2018; Mills Ko et al., 2014; Niu et al., 2019). Therefore, our finding of reduced CXCR3+ CD4+ T cell infiltration into the CNS of mice with EAE is consistent with but not exclusively due to lack of interaction between endothelial cell Cav-1 and T cell CXCR3.

In conclusion, our studies demonstrate that the importance of CXCL10 on T cell infiltration might be context dependent. This is highlighted by the fact that Cav-1 deficiency did not fully abrogate neuroinflammation and neurological signs in the EAE model. Our data suggest that while endothelial Cav-1 promotes transcellular migration of CXCR3+ T cells across the BBB, paracellular migration contributes to neuroinflammation in the absence of Cav-1. This is also consistent with reports indicating that strategies to manipulate CXCL10/CXCR3 expression and function in EAE variably do or do not attenuate clinical and neuropathological signs of disease (Heng et al., 2022; Karin, 2020; Klein et al., 2004; Kohler et al., 2008; Lalor & Segal, 2013; Mills Ko et al., 2014; Sporici & Issekutz, 2010). Thus, a further limitation of this study is that the translational significance of CXCL10/Cav-1 signaling pathway is tempered by the fact that it is only one of many signaling interactions happening at the BBB. Future studies can test if the contribution of the CXCL10/CXCR3/Cav-1 axis to neuroinflammation may be greater in other neuroinflammatory diseases associated with robust IFNγ inducible CXCL10 such as SARS-CoV-2 (Blank et al., 2016; Greene et al., 2024; Sorensen et al., 2018). The potential importance of this pathway for post-infectious neuroinflammation is underscored by a previous report that brain-endothelial derived CXCL10 mediates behavioral changes through impairment of synaptic plasticity (Blank et al., 2016). Overall, our findings establish a novel mechanism by which BEC present CXCL10 to license specific T cell subsets for transcellular migration across the BBB.

## Supplementary Material

CXCL10 manuscript supplementary R2.pdf

CXCL10 manuscript supplementary statistical information V2.xlsx
