# Supplementary material for: A Brain Endothelial Cell Caveolin-1/CXCL10 Axis Promotes T Cell Transcellular Migration Across the Blood-Brain Barrier
Source: ASN Neuro. 2025 Mar 10;17(1):2472070. doi: 10.1080/17590914.2025.2472070 (PMC12047051; doi:10.1080/17590914.2025.2472070)
Supplement: CXCL10 manuscript supplementary R2.pdf [file TASN_A_2472070_SM8532.pdf]

A brain endothelial cell Caveolin-1/CXCL10 axis promotes T cell transcellular migration across the blood-brain barrier

Supplemental Figures:

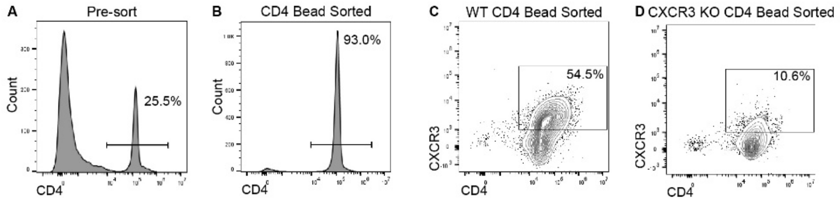

Supplementary Figure 1: CXCR3+ CD4+ T cell enrichment in culture. A) CD4+ T cell percentage of total culture after 5 days *in vitro* (DIV). Gated on live cells. **B)** CD4+ T cell percentage after sorting with MojoSort CD4 Beads. Gated on live cells. **C)** CXCR3+ CD4+ T cells were assessed from WT Th1 cultures at 5 DIV after sorting with MojoSort CD4 Beads.

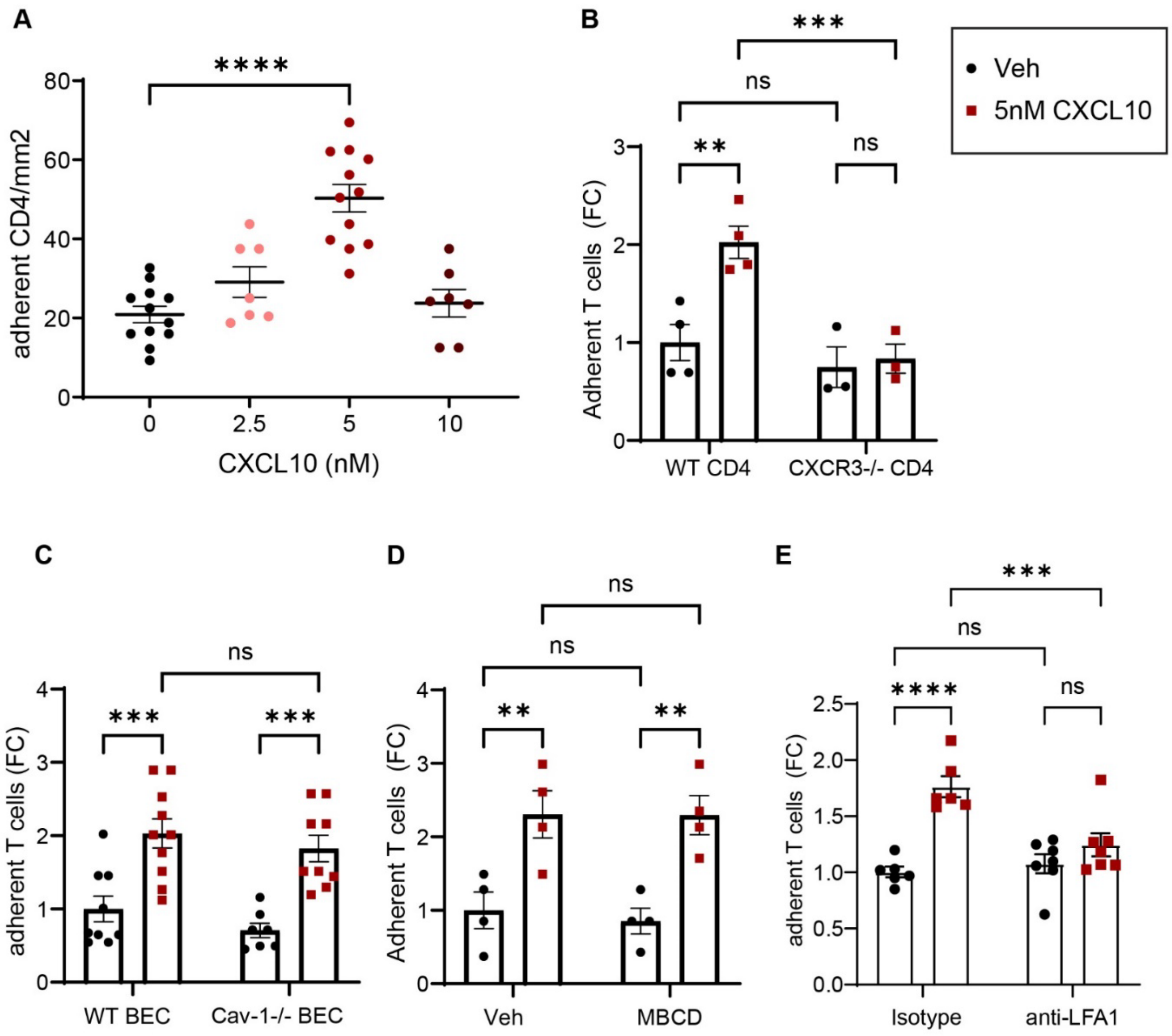

Supplementary Figure 2. CXCL10 increases T cell adhesion to BECs. A) T cell adhesion to BEC monolayer is influenced by CXCL10. CXCL10 was applied to the T cell/BEC coculture in the indicated concentrations. One-way ANOVA indicated significant effect of concentration [F = 6.5339, p = 0.0043]. Dunnett's multiple comparisons test showed that 5nM CXCL10 significantly increased CD4+ T cell adhesion to BECs as compared to vehicle treatment (p = 0.0024).

B) Adhesion of WT and CXCR3 KO CD4+ T cells to mBEC, with 5 nM CXCL10 or vehicle. Points represent averages from 3-4 independent experiments. Adherent cell count includes migratory CD4+ T cells. Two way ANOVA demonstrated significant effect of CXCL10 [$F_{(1, 10)}$ = 9.459, p = 0.0117], genotype [$F_{(1, 10)}$ = 15.91, p = 0.0026], and interaction [$F_{(1, 10)}$ = 6.770, p = 0.0264]. Fisher's LSD test revealed that WT CD4+ T cells

stimulated with CXCL10 adhered significantly more than did WT T cells without CXCL10 (p = 0.0015), and significantly more than T cells deficient in CXCR3 (p = 0.0009).

C) Quantitation of CD4+ T cells adherent to WT and Cav-1$^{-/-}$ mBECs in the presence of 5 nM CXCL10 or vehicle. Points represent averages from 4-7 independent experiments. Adherent cell count includes migratory CD4+ T cells. Two way ANOVA demonstrated significant effect of CXCL10 [$F_{(1, 31)} = 35.96$, $p < 0.0001$]. Fisher's LSD revealed that CXCL10 increased T cell adhesion to WT BEC (p = 0.0002) and to Cav-1$^{-/-}$ BEC (p = 0.0002).

D) Quantitation of CD4+ T cell adhesion to WT and MβCD-treated mBEC with 5 nM CXCL10 or vehicle. Points represent averages from 4 independent experiments. Adherent cell count includes migratory CD4+ T cells. Two way ANOVA demonstrated significant effect of CXCL10 [$F_{(1, 12)} = 28.28$, $p = 0.0002$]. Fisher's LSD revealed that CXCL10 increased T cell adhesion to BEC treated with MβCD (p = 0.002) and to BEC treated with vehicle (p = 0.0038).

E) Quantification of adhesion of anti-LFA-1 treated or isotype-control treated CD4+ T cells to mBEC in the presence of 5 nM CXCL10 or vehicle. Points represent the average from 7 experiments. Adherent cell count includes migratory CD4+ T cells. Two-way ANOVA demonstrated significant effect of CXCL10 [$F_{(1, 20)} = 4.770$, $p = 0.0410$], anti-LFA-1 antibody [$F_{(1, 20)} = 5.621$, $p = 0.0279$] and CXCL10*anti-LFA-1 interaction [$F_{(1, 20)} = 11.19$, $p = 0.0032$]. Fisher's LSD test revealed that CXCL10 increased adhesion of isotype-control treated T cells (p = 0.0017) but not of anti-LFA-1 treated T cells (p = 0.3789). anti-LFA-1 treated T cells had significantly less adhesion than did isotype-control treated T cells in response to CXCL10 (p = 0.0043).

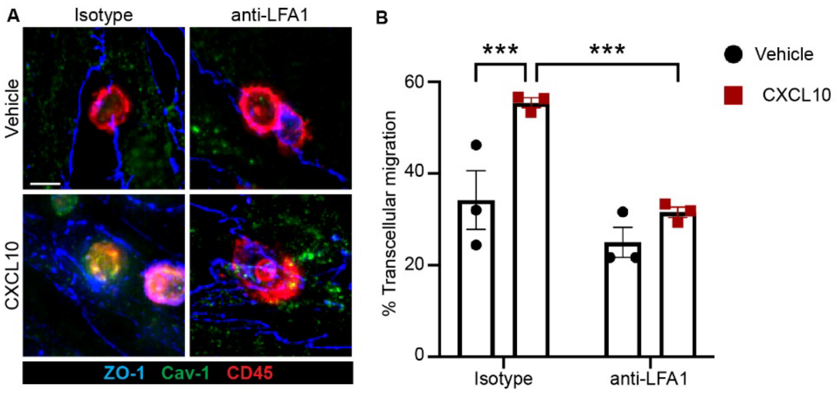

Supplementary Figure 3. LFA-1 contributes to CXCL10-induced transcellular transmigration. A) Representative images of isotype or anti-LFA-1 treated CD4+ T cells transmigrating across mBECs in the presence of 5 nM CXCL10 or vehicle. Immunostaining was conducted for junctional ZO-1 (blue), Cav-1 (green), and CD45 (red). Scale bar, 10 µm. **B)** Relative frequency of transcellular migration of isotype or anti-LFA-1 treated CD4+ T cells across mBECs in the presence of 5 nM CXCL10 or vehicle. Points represent averages from 3 independent experiments. Two way ANOVA demonstrated significant effect of CXCL10 [$F_{(1, 8)} = 10.22$, $p = 0.0127$] and interaction between LFA-1 blockade and CXCL10 [$F_{(1, 8)} = 12.84$, $p = 0.0072$]. Fisher's LSD revealed that CXCL10 significantly increased transmigration of isotype-control treated T cells ($p = 0.0014$) but not anti-LFA-1 treated T cells ($p = 0.256$). Anti-LFA-1 treated T cells migrated significantly less than did isotype-control treated T cells in response to CXCL10 ($p = 0.0049$).

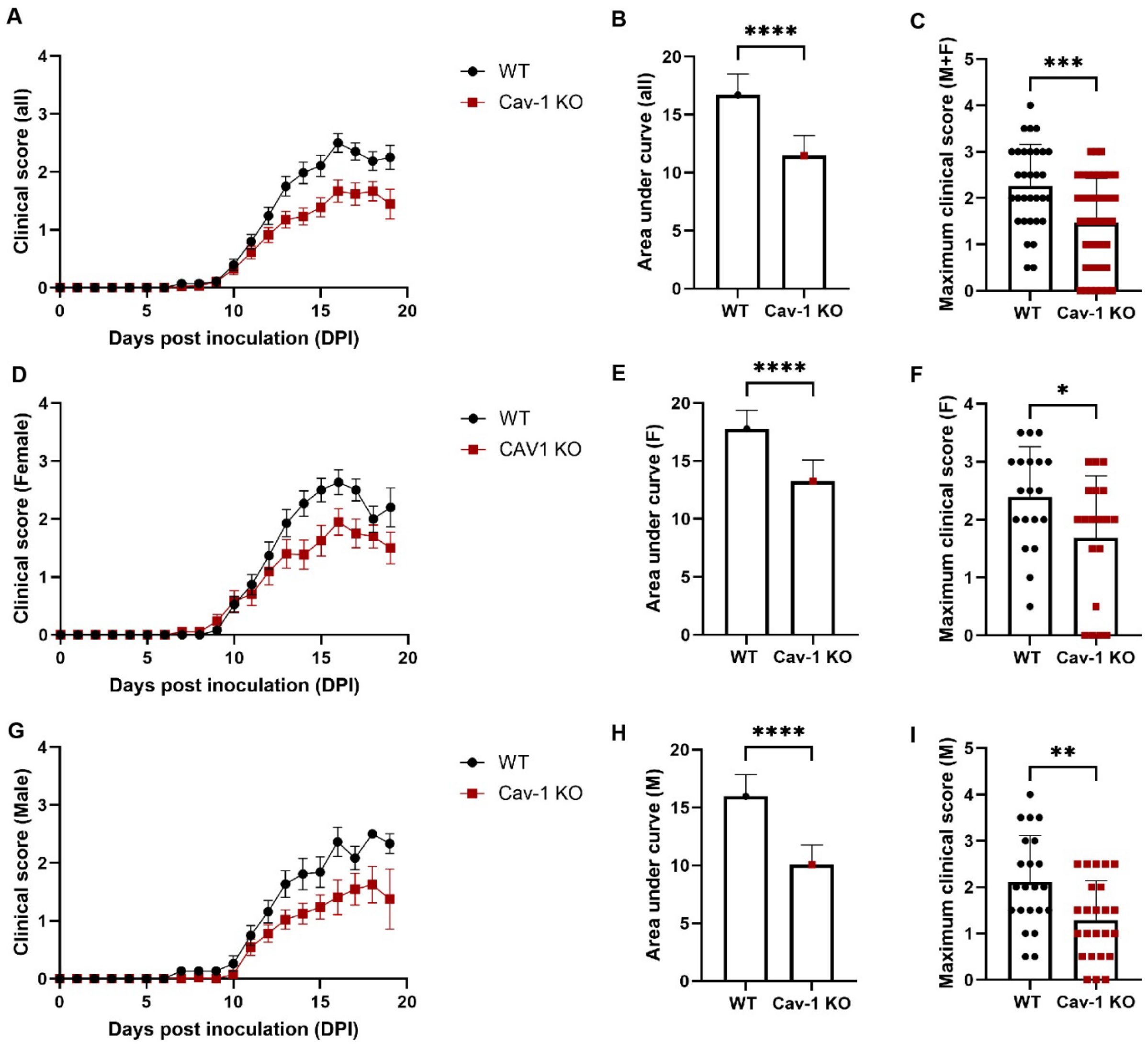

Supplementary Figure 4. Cav-1$^{-/-}$ female and male mice have less severe EAE clinical signs than WT. A) Clinical curve of EAE in female and male WT (n=41) and Cav-1$^{-/-}$ (n=43) mice from 6 independent experiments. **B)** Area under the curve analysis of female and male WT and Cav-1$^{-/-}$ clinical curves for 0-19 DPI. Unpaired t-test, p <0.0001. **C)** Peak clinical score is significantly lower in female and male Cav-1$^{-/-}$ than in WT EAE. Mann-Whitney t-test, p = 0.0009. **D)** Clinical curve of EAE in female WT (n=19) and Cav-1-/- (n=19) mice from 6 independent experiments. **E)** Area under the curve analysis of female WT and Cav-1$^{-/-}$ clinical curves for 0-19DPI. Unpaired student t-test, p <0.0001. **F)** Peak clinical score is significantly lower in female Cav-1$^{-/-}$ than in WT EAE. Mann-Whitney t-test, p = 0.04. **G)** Clinical curve of EAE in male WT (n=22) and Cav-1$^{-/-}$ (n=24)

mice from 6 independent experiments. **H)** Area under the curve analysis of male WT and Cav-1$^{-/-}$ clinical curves for 0-19DPI. Unpaired student t-test, p <0.0001. **I)** Peak clinical score is significantly lower in male Cav-1$^{-/-}$ than in WT EAE. Mann-Whitney t-test, p = 0.0079.

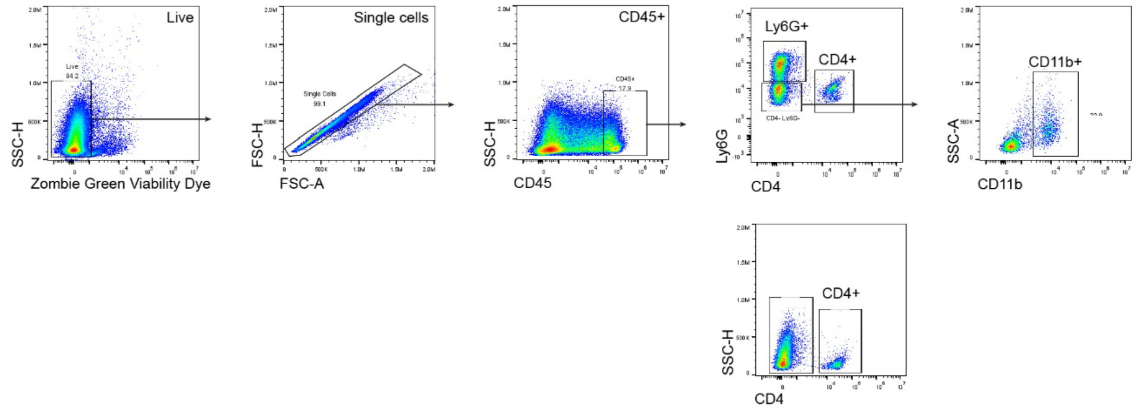

Supplementary Figure 5. Flow cytometry gating strategy. Sequential gates were applied to select for CNS leukocyte live populations (based on negativity for the Zombie green viability dye), singlets (based on forward scatter height:area), and CD45^{hi} (as a leukocyte marker). To assess T cells, CD45+ cells were gated for CD4. CD4+ cells were then gated for CXCR3 (Figure 3). Additional characterization of CD4-, CD45+ cells included gating for Ly6G and CD11b.

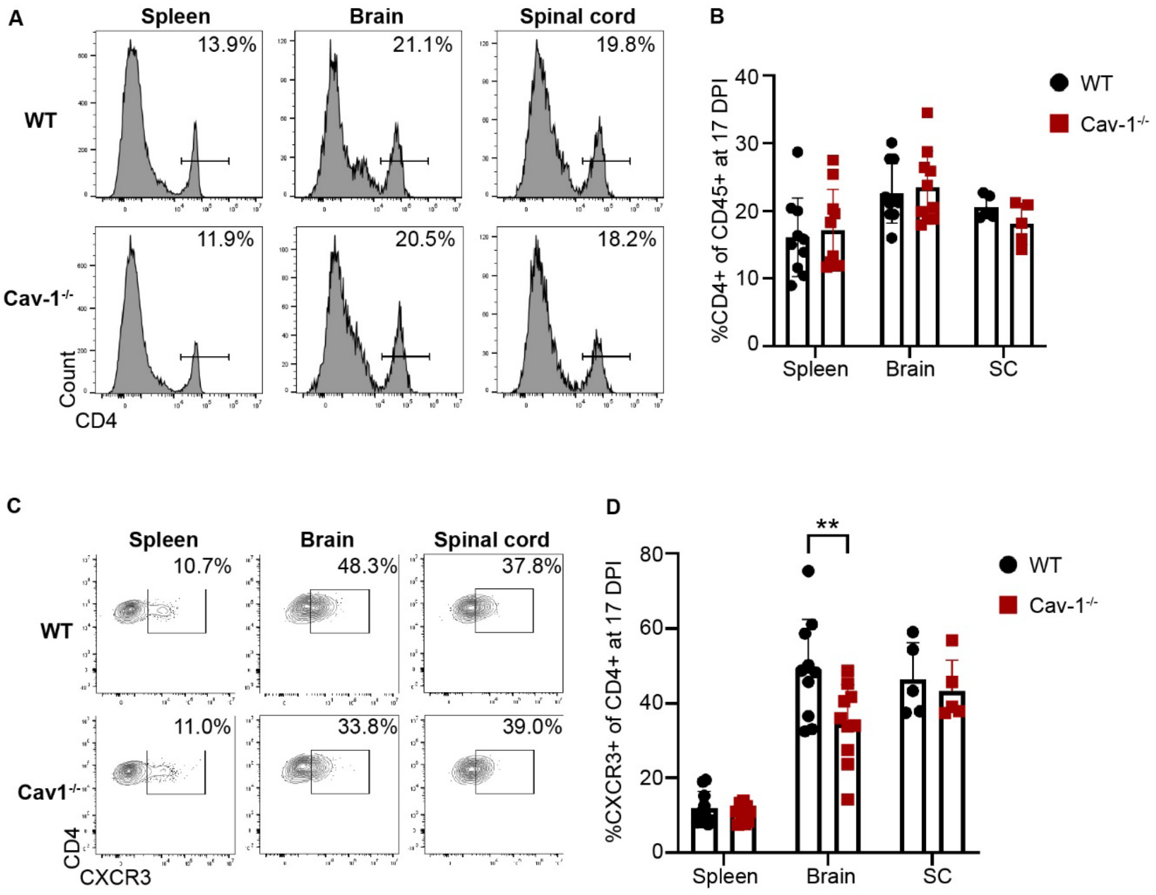

Supplementary Figure 6. Cav-1 contributes to CXCR3+ CD4+ T cell infiltration of the CNS at the peak of acute clinical signs of disease at 17DPI. A) Representative histograms of flow cytometry analysis of CD4+ population in spleen, brain, and spinal cord (SC) of WT and Cav-1$^{-/-}$ mice during peak acute EAE. **B)** Quantification of viable CD4+, CD45+ cells in flow cytometry analysis of spleen, brain, and spinal cord from WT and Cav-1$^{-/-}$ mice during peak acute EAE. **C)** Representative plots of flow cytometry analysis of CXCR3+ CD4+ cells in the population of viable CD45+ cells from spleen, brain, and spinal cord of WT and Cav-1$^{-/-}$ mice during peak acute EAE. **D)** Quantification of CXCR3+ CD4+ population of viable, CD45+ cells in flow cytometry analysis of spleen, brain, and spinal cord from WT and Cav-1$^{-/-}$ mice during peak acute EAE. Two-way ANOVA demonstrated significant effect of genotype [$F_{(1, 44)} = 5.584$, $p = 0.0226$] and organ [$F_{(2, 44)} = 75.63$, $p < 0.0001$]. Sidak's multiple comparison test revealed significantly

fewer CXCR3+ CD4+ T cells in the brain of Cav-1$^{-/-}$ mice with EAE as compared to the brain of WT mice with EAE (p = 0.0022).

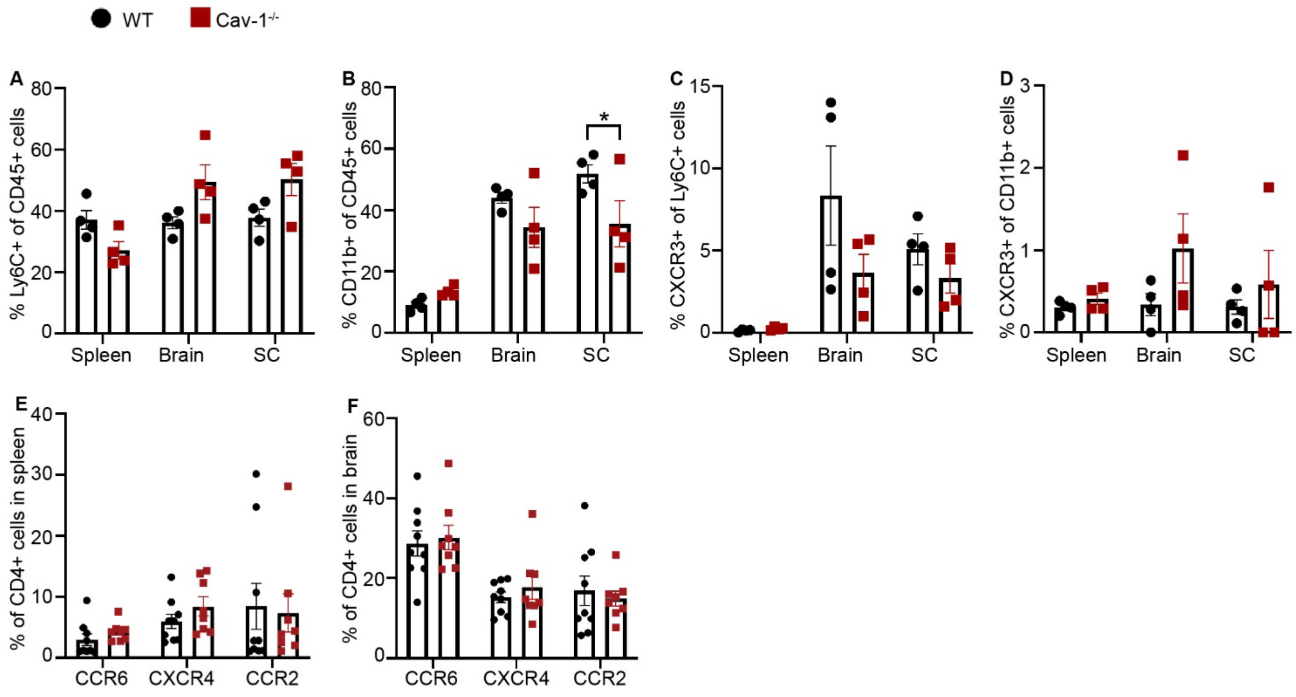

Supplementary Figure 7. Cav-1$^{-/-}$ and WT mice have similar frequencies of innate immune cells and CD4 T cells with indicated chemokine receptor expression near the onset of clinical signs of EAE at 10 DPI. A) Quantification of Ly6C+ population of viable, CD45+ cells in flow cytometric analysis of spleen, brain, and spinal cord (SC) from WT and Cav-1$^{-/-}$ mice during EAE onset. **B)** Quantification of CD11b+ population of viable, CD45+ cells in flow cytometry analysis of spleen, brain, and spinal cord from WT and Cav-1$^{-/-}$ mice during EAE onset. **C)** Quantification of CXCR3+ Ly6C+ population of viable, CD45+ cells in flow cytometry analysis of spleen, brain, and spinal cord from WT and Cav-1$^{-/-}$ mice during EAE onset. **D)** Quantification of CXCR3+ CD11b+ population of viable, CD45+ cells in flow cytometry analysis of spleen, brain, and spinal cord from WT and Cav-1$^{-/-}$ mice during EAE onset. **E)** Chemokine receptor analysis of CD45+ cells from spleen of WT and Cav-1$^{-/-}$ mice during EAE onset. **F)** CCR6, CXCR4, and CCR2 chemokine receptor positive CNS-infiltrating leukocytes from WT and Cav-1$^{-/-}$ mice during EAE onset. Two-way ANOVA with Sidak's post-hoc comparisons between WT and Cav-1$^{-/-}$ groups. * $p<0.05$.
